# The Lantibiotic NAI-107 Efficiently Rescues Drosophila melanogaster from Infection with Methicillin-Resistant Staphylococcus aureus USA300

**DOI:** 10.1128/AAC.02965-15

**Published:** 2016-08-22

**Authors:** Thomas T. Thomsen, Biljana Mojsoska, João C. S. Cruz, Stefano Donadio, Håvard Jenssen, Anders Løbner-Olesen, Kim Rewitz

**Affiliations:** a Department of Biology, University of Copenhagen, Copenhagen, Denmark; b Department of Science, Systems and Models, Roskilde University, Roskilde, Denmark; c Ktedogen, Milan, Italy; d Naicons Srl, Milan, Italy

## Abstract

We used the fruit fly Drosophila melanogaster as a cost-effective *in vivo* model to evaluate the efficacy of novel antibacterial peptides and peptoids for treatment of methicillin-resistant Staphylococcus aureus (MRSA) infections. A panel of peptides with known antibacterial activity *in vitro* and/or *in vivo* was tested in Drosophila. Although most peptides and peptoids that were effective *in vitro* failed to rescue lethal effects of S. aureus infections *in vivo*, we found that two lantibiotics, nisin and NAI-107, rescued adult flies from fatal infections. Furthermore, NAI-107 rescued mortality of infection with the MRSA strain USA300 with an efficacy equivalent to that of vancomycin, a widely applied antibiotic for the treatment of serious MRSA infections. These results establish Drosophila as a useful model for *in vivo* drug evaluation of antibacterial peptides.

## INTRODUCTION

Since the golden era of antibiotic drug development of the 1940s to 1960s, the development and spread of multidrug resistance (MDR) have become a huge burden to societies. At present, resistance to almost all known antibiotics has emerged with the sequential introduction of new or improved antibiotics in clinical and agricultural settings (1, 2). Therefore, continued development of new or improved antibiotics is of great importance to human health. However, new antibiotics are lacking, and few are under development for treatment of MDR infectious bacteria, as drug development is costly and success from *in vitro* discovery to application in clinical settings is limited.

Bacterial infections with methicillin-resistant Staphylococcus aureus (MRSA) are no longer sporadic in their distribution and prevalence (3, 4). MRSA strains are associated with both community (CA-MRSA)- and hospital (HA-MRSA)-acquired infections, with the highly β-lactam-resistant CA-MRSA clone USA300 accounting for up to 80% of all MRSA infections in the United States (5). High-level β-lactam resistance is due to acquisition of staphylococcal cassette chromosome *mec* (SCC*mec*) elements, including the *mecA* gene, which encodes an alternative version of the penicillin binding protein (PBP2A) that is inducible (6, 7) and has a lowered affinity for β-lactam antibiotics (8). SCC*mec* elements are often associated with carriage of genes encoding products involved in resistance to other antibiotics, including aminoglycoside-modifying enzymes, such as acetyltransferase, adenylyltransferase, or phosphotransferase (9). Due to this resistance, MRSA treatment often includes glycopeptide antibiotics, such as vancomycin, or oxazolidinones, such as linezolid. However, failure of vancomycin treatment has been reported for vancomycin-intermediate S. aureus (VISA) (10) and vancomycin-resistant S. aureus (VRSA) (11) strains. On the other hand, linezolid resistance is rare (12) but has been observed in association with mutations in the rRNA gene encoding the 23S RNA or through carriage of a Cfr rRNA methyltransferase gene (13, 14). Furthermore, resistance to the last-resort antibiotic daptomycin has been reported (15, 16). Given the increasing frequency of resistance to these antibiotics, it is important to develop improved or novel therapeutics and to consider new strategies to contain the spread of the growing resistance problem.

Peptide-based antibiotics have been proposed as the next generation of antibacterial compounds because of their widespread distribution in nature as part of innate immunity. These molecules are often amphipathic and interact with the bacterial membrane to disrupt its function. The cationic peptide colistin, a bacteriocin currently used for treatment of highly resistant Gram-negative infections, is part of the polymyxins that are derived from natural producers, such as Paenibacillus polymyxa (17). Another bacteriocin, nisin, has for decades been used in the food industry against harmful bacteria, such as S. aureus, Listeria monocytogenes, and Clostridium botulinum (18). Nisin belongs to a subgroup known as lantibiotics, named so for containing uncommon amino acids, such as lanthionine, methyllanthionine, didehydroalanine, or didehydroaminobutyric acid (19). Nisin has been described to disrupt membrane integrity through a dual mode of action, with inhibition of cell wall synthesis by binding to the cell wall precursor lipid II and subsequent pore formation (20 – 22), although new evidence points toward a more complex mechanism that includes aggregation of lipid II (23). Peptides may be used directly as antibacterials or can serve as templates for development of small-molecule mimetics, such as peptoids, which can accommodate improvements to toxicity and are intrinsically less prone to degradation by proteases (24).

The gap from *in vitro* drug screening to the large-scale efficacy testing necessary for clinical development is hampered by the expensive, labor-intensive, and highly regulated mammalian infection models. It is therefore of interest to develop improved, cost-effective methods with high predictive value for screening of antibacterial compounds before these are tested in mammalian models. Although the fruit fly Drosophila melanogaster has been used in drug discovery (25, 26), its application for screening of antibacterial compounds has been limited (27 – 29). Drosophila is a powerful genetic model for studying disease mechanisms, and during the past decades, it has been used extensively in elucidating the mechanisms of innate immunity, leading to the discovery of the conserved role of the Toll receptors (30) and the immune deficiency (IMD) pathway (31). Studies of innate immunity in Drosophila have sprouted the development of various methods for infecting flies with important human pathogens (28, 32 – 34). In the present study, we evaluated the therapeutic potential of antibacterial peptides and peptoids *in vivo* by testing their efficacy in a Drosophila model of infection with S. aureus 8325-4 (35) or MRSA USA300 (36). Tests were performed with a range of different peptides, including the lantibiotics nisin A (nisin) (37) and NAI-107 (38, 39), a compound that is currently undergoing preclinical studies. Lantibiotics are usually produced by Gram-positive bacteria and are characterized as ribosomally synthesized peptides containing posttranslationally created ring structures introduced through a thioether containing lanthionine and methyllanthionine residues (40). Furthermore, the following panel of synthesized amphipathic cationic peptides previously shown to have good *in vitro* and/or *in vivo* efficacy were tested: GN-2 and -4 (41, 42), HHC-9 (43), HHC-36 (44), and the peptoids GN-2 Npm_9_, GN-2 Ntrp_5–8_, and GN-4 (45). We found that NAI-107 rescued an otherwise lethal infection with MRSA USA300 with an efficacy equivalent to that of vancomycin in Drosophila. Our findings also show that treatment with nisin extends life expectancy in animals infected with MRSA USA300, while the majority of the peptides and peptoids tested showed no protection from infection or had detrimental effects on the survival of the host.

## MATERIALS AND METHODS

### Bacteria and growth media.

S. aureus strains 8325-4 (35) and USA300 (36) were used as indicated for the individual experiments. Bacterial cultures were grown in cation-adjusted Mueller-Hinton broth (MHB-II) at the indicated temperature.

### Growth rates and determination of bacterial loads.

The growth rates of S. aureus strains were examined at 37°C *in vitro* to determine the growth period required to obtain balanced cultures, here defined as cultures grown exponentially for no fewer than 6 generations. Prior to injection of bacteria into the *in vivo* fly model, the inoculums were prepared as balanced cultures grown at 37°C. Since flies used for *in vivo* infections were kept at 29°C, the bacterial *in vitro* generation time was also determined at this temperature. *In vitro* growth rates were defined for growth in MHB-II by measurements of the optical density at 600 nm (OD_600_) at 10-min intervals. Furthermore, we determined the *in vivo* generation time based on CFU per animal by counting the CFU at various time points by homogenizing flies infected with bacteria and plating the homogenates on mannitol salt agar (MSA). This was performed in triplicate experiments; 3 individual flies were crushed in phosphate-buffered saline (PBS), and 10× dilution series were prepared, from which 10 μl each was spot plated on MSA in triplicate. The mean value for each experiment was determined as the number of CFU per fly and plotted. However, for determination of bacterial titers posttreatment, four replicative experiments with 40 flies each were performed, with 8 live individual flies homogenized at each time point. This homogenate was diluted in 1 ml PBS, and dilutions were plated as 100-μl aliquots on MSA at 0, 3, 12, and 24 h. It should be noted that the concentration of drug inside each fly was based on multiples of the MIC in 0.5 μl fly liquid content (described in the next section). Therefore, it was not necessary to further wash the homogenate, as the drug inside the fly could be estimated to be diluted approximately 1:2,000. Finally, initial trials showed that washing of the homogenate provided lower CFU titers than those obtained with unwashed samples. It should also be noted that drug treatment was performed at 3 h postinfection and that the number of CFU per fly for this time point was determined prior to treatment.

### MIC testing.

MICs of all tested compounds were determined according to protocols using broth microdilution methodology (46), with minor modifications. S. aureus was grown in 10 ml MHB-II overnight at 37°C with shaking and then diluted 1:100 in fresh MHB-II and grown to an OD_600_ of 0.2 to 0.4. Cultures were then diluted 1:10 and grown to an OD_600_ of 0.2 to 0.4. These steps were performed to ensure balanced exponentially growing cultures as explained above. Finally, cultures were diluted to 1 × 10^6^ CFU ml^−1^ and further diluted 1:1 in microtiter plates in MHB-II with drug, leading to a final inoculum of 5 × 10^5^ CFU ml^−1^. MICs were determined in triplicate, and if more than one value was found, the highest was set as the MIC to keep the calculations conservative.

### Injection assay.

Injection assays were performed as previously described (33) by using a Nanoject-II microinjecter, but with minor modifications in the preparation of bacterial inoculums to obtain balanced cultures, as explained above. Flies were reared on standard Bloomington formulation at 25°C under a 12-h–12-h light-dark cycle and constant humidity. Adult Oregon R male flies (4 to 7 days old) were used for all injection experiments. Initial experiments with strain 8325-4 were performed in duplicate, with groups of 25 to 30 animals in each experiment. For USA300, experiments were performed in triplicate, except for the nisin experiment, which was done only in duplicate. The inoculum was prepared by resuspending cells in 10 mM MgSO_4_ vehicle (VEH) to an OD_600_ of 0.06 and was kept on ice, giving an inoculum dose of 100 to 450 CFU (8325-4) or 200 to 700 CFU (USA300) in the flies after injection of 18.4 nl. Bacterial and VEH injections were administered in the soft tissue surrounding the front legs, and drug treatment was administered in the lower thorax at 3 h postinfection. After injection of bacteria, flies were kept at 29°C and monitored for 48 to 96 h to determine mortality. Drug delivery was performed at the concentrations indicated for individual experiments. Flies which died within 3 h postinjection were considered to have died from handling and were disregarded. It is important that when drug concentrations were calculated, we performed a rough approximation of the fluid content of a fly. Fly fluid content was measured by drying out 10 groups of 50 flies each and comparing dry weight to wet weight. This resulted in an average fluid content of 0.58 μl per adult male fly. For simplicity and because we assumed that the compounds would not be distributed to all fluids, we used 0.5 μl fluid as our measure for calculating drug concentrations in the flies. Further, we assumed a rapid distribution of the compound in the open circulatory system of Drosophila and a slow clearance of the compounds by Malpighian tubules. Therefore, drug concentrations are given as the highest concentrations obtained, in multiples of the MIC.

### Statistics and graphical plots.

Plotting of data was performed using GraphPad Prism 5. All *in vivo* survival plots were created using Kaplan-Meier analysis of pooled data for repetitive experiments. Statistical analysis was carried out with the log rank (Mantel-Cox) test for comparison of survival curves. Experiments with *P* values of <0.05 were considered to be significant and are stated in Results. Statistical analysis was performed on the counts of CFU per fly for the different treatment groups by using one-way analysis of variance (ANOVA) (Kruskal-Wallis test) with Dunn's multiple-comparison test for comparison of individual groups. Relevant statistical results are stated in Results.

### RNA preparation and qPCR.

Isolation of total RNA for quantitative PCR (qPCR) was performed by use of an RNeasy minikit (Qiagen) according to the manufacturer's instructions. Biological samples were collected as 10 adult male flies pooled for each replicate and time point. To reduce contamination with genomic DNA, all samples were treated on-column with DNase. Total RNA concentrations were measured on a Qubit 3.0 fluorometer, and equivalent amounts of total RNA were used for cDNA synthesis for each sample. cDNA synthesis was performed using a SuperScript III first-strand synthesis kit (Invitrogen) according to the manufacturer's instructions. qPCR was performed on an Mx3000P qPCR system (Agilent Technologies) using the following program: 95°C for 10 min followed by 45 cycles of 95°C for 15 s, 60°C for 15 s, and 72°C for 15 s. Dissociation curve analysis was applied to all reactions. Primers are described in Table S1 in the supplemental material. We used *Rpl23* as a housekeeping gene to normalize expression as previously described (47).

### Compounds.

Ampicillin sodium salt (99%; Roth) was used as a control for efficacy in *in vitro* and *in vivo* experiments. Vancomycin was acquired from Hospira as vancomycin hydrochloride for intravenous treatment. The peptides GN-2, GN-4, HHC-9, and HHC-36 (all amidated at the C terminus), nisin A (nisin), and peptoids were above 95% purity and were synthesized and/or purified by Håvard Jenssen, Roskilde University, Denmark. NAI-107 is a complex of congeners produced by Microbispora sp. 107891 and was prepared as previously described (48). The distribution of congeners for the batch used in the current study was as follows: A1 + A2 = 80.8%, F1 + F2 = 9.4%, and B1 + B2 = 4%.

## RESULTS

### Determination of growth of S. aureus in vitro and *in vivo* in a Drosophila infection model.

**FIG 1:**
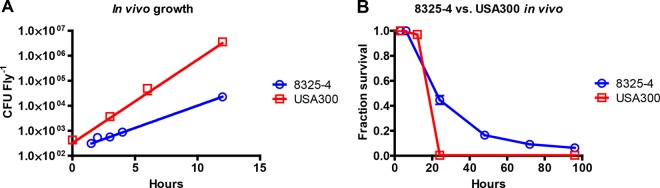
*In vivo* growth rates and killing of flies of the two bacterial isolates. (A) The *in vivo* growth rate of USA300 was 54 min, and that of strain 8325-4 was 104 min, demonstrating a difference in proliferation. Three flies were homogenized and serial dilutions were made in PBS before plating on S. aureus-selective MSA to determine the number of CFU. (B) USA300 killed close to 100% of infected flies within 24 h, while isolate 8325-4 killed approximately 50% of flies within 24 h of infection (*P* < 0.0001). Minor differences in the starting inoculums were observed (see Materials and Methods). Survival data are compiled results from all *in vivo* kill rate experiments presented in Fig. 2 and 4.

We determined the growth rates of S. aureus strain 8325-4 and MRSA strain USA300 in MHB-II medium at 29°C because all successive *in vivo* experiments were performed at this temperature. Strain 8325-4 had a generation time of 57 min, while USA300 had a generation time of 44 min (data not shown). The *in vivo* growth rates of the same strains were determined by injection of bacteria into flies at time zero, with samples collected at time zero and at 3, 4 to 6, and 12 h postinfection. USA300 had a generation time of 54 min, whereas 8325-4 had a generation time of 104 min *in vivo* (Fig. 1A). Drosophila flies infected with USA300 died rapidly, with no surviving flies at 24 h postinfection (Fig. 1B). Flies infected with approximately the same number of 8325-4 organisms lived significantly longer. We suggest that this difference in viability reflects the difference in *in vivo* growth rates of USA300 and 8325-4 bacteria.

### MICs of antibacterial peptides and peptoids.

We determined the MICs of peptides and peptoids for the two strains (Table 1). The MIC values of the amphipathic cationic peptides GN-2, GN-4, HHC-9, and HHC-36 and the lantibiotic nisin for S. aureus 8325-4 were in the range of 4 to 10 μg ml^−1^, while those of the GN-2 and GN-4 peptoids were higher (16 to 64 μg ml^−1^). On the other hand, the MIC of NAI-107 against strain 8325-4 was only 0.06 μg ml^−1^, showing that NAI-107 is highly efficient at inhibiting *in vitro* growth of S. aureus. The MIC of NAI-107 for S. aureus 8325-4 was comparable to that of ampicillin (0.01 μg ml^−1^). For S. aureus USA300, the MIC was 0.25 μg ml^−1^ for NAI-107 and 2 μg ml^−1^ for vancomycin.

**TABLE 1:** MICs of compounds tested^*a*^

Compound	Sequence (N terminus-C terminus)	Stock solute	mol wt	MIC (μg ml^−1^)		MIC (μM)	
				8325-4	USA300	8325-4	USA300
Peptides							
Nisin		H_2_O	3,354	10	4	2.98	1.19
NAI-107		DMSO	2,238	0.06	0.25	0.03	0.11
GN-2	RWKRWWRWI-CONH_2_	H_2_O	1,473	8	NA	5.43	NA
GN-4	RWKKWWRWL-CONH_2_	H_2_O	1,445	4	NA	2.77	NA
HHC-9	RWRRWKWWL-CONH_2_	H_2_O	1,473	4	NA	2.71	NA
HHC-36	KRWWKWWRR-CONH_2_	H_2_O	1,488	8	NA	5.38	NA
Peptoids							
GN-2 Npm_9_	H-*N*ae-*N*trp-*N*ae-*N*ae-*N*trp-*N*trp-*N*ae-*N*trp-*N*ile-NH_2_	H_2_O	1,477	32	NA	22	NA
GN-2Ntrp_5–8_	H-*N*lys-*N*lys-*N*lys-*N*lys-*N*trp-*N*trp-*N*trp-*N*trp-NH_2_	H_2_O	1,443	16	NA	11	NA
GN-4	H-*N*lys-*N*trp-*N*lys-*N*lys-*N*trp-*N*lys-*N*trp-*N*leu-NH_2_	H_2_O	1,443	64	NA	44	NA
Control antibiotics							
Ampicillin		H_2_O	349	0.01	NA	2.86	NA
Vancomycin		H_2_O	1,449	NA	2	NA	1.38

a The molecular weights used for calculations of micromolar concentrations are given in the table. MICs of some compounds were not determined for both isolates (NA). Sequences of nisin and NAI-107 are not included because they contain ring structures, making linear sequences misleading. Most compounds were dissolved in H_2_O. However, the NAI-107 stock solution was prepared in 100% dimethyl sulfoxide (DMSO), but experimental *in vitro* and *in vivo* concentrations of DSMO did not exceed 0.5%, except in *in vivo* experiments with NAI-107 at 100× MIC (∼6% DMSO). However, injection of 18.4 nl into a fly containing ∼500 nl fluid causes rapid dilution of the DMSO.

### Identification of nisin and NAI-107 as efficacious treatments for systemic S. aureus infections in a Drosophila in vivo model.

**FIG 2:**
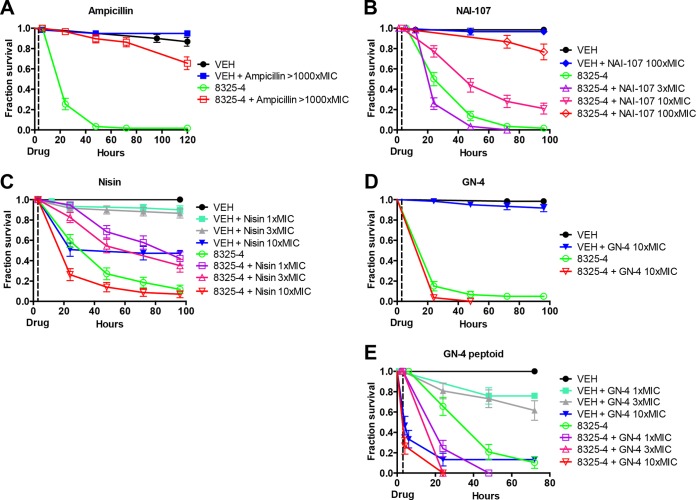
*In vivo* efficacies of compounds against S. aureus 8325-4 in a Drosophila whole-animal model. The graphs show the effects of different peptides and peptoids on survival of flies. (A) Ampicillin; (B) NAI-107; (C) nisin; (D) GN-4; (E) GN-4 peptoid. Flies were counted at 0, 3, 6, 12, 24, and 48 to 120 h. Flies were injected with either vehicle (VEH) or isolate 8325-4 at time zero, with the indicated treatments given at 3 h (dotted lines). Flies were counted prior to injection with compound. Compound concentrations are given as approximate concentrations in animals.

To evaluate the therapeutic potential of the peptides and peptoids, we determined their ability to rescue flies with an otherwise lethal systemic S. aureus 8325-4 infection. In order to establish the appropriate dosages, we used the following reasoning. Because insects are known to have an open circulatory system, we assumed that the administered compound would be distributed rapidly and uniformly in the hemolymph of the fly. The volume of the fly hemolymph was estimated to be 0.5 μl (see Materials and Methods), and we assumed that clearance was slow. With these assumptions, the highest concentration achieved for each compound can be expressed as a multiple of the MIC. For example, for nisin, 1× MIC (10 μg ml^−1^) is equivalent to injection of 2.5 mg nisin kg fly^−1^, and these calculations can be found in Table 2 for all drugs. Ampicillin was chosen as a control, as β-lactams in general are considered nontoxic to the host and can be administered at high concentrations, in our case >1,000× MIC. Ampicillin efficiently promoted survival of 8325-4-infected flies (*P* < 0.001) (Fig. 2A) over a 70-h period, with no lethal effects on control animals (*P* = 0.15), here defined as no difference in survival between flies injected with VEH and those injected with both VEH and drug.

**TABLE 2:** Antibacterial peptide dosages^*a*^

Compound	Target 1× MIC (μg μl^−1^) in fly		amt injected for 1× MIC (μg)		amt of compound injected (mg kg fly^−1^)	
	8325-4	USA300	8325-4	USA300	8325-4	USA300
Peptides						
Nisin	0.01	0.004	0.005	0.002	6.25	2.5
NAI-107	0.00006	0.00025	0.00003	0.000125	0.04	0.16
GN-2	0.008		0.004		4	
GN-4	0.004		0.002		2.5	
HHC-9	0.004		0.002		2.5	
HHC-36	0.008		0.004		5	
Peptoids						
GN-2 Npm_9_	0.032		0.016		20	
GN-2 Ntrp_5–8_	0.016		0.008		10	
GN-4	0.064		0.032		4	
Control antibiotics						
Ampicillin	0.00001		0.000005		0.01	
Vancomycin		0.002		0.001		1.25

a All data presented are based on 1× MICs of the compounds. The fly weight was 0.8 mg, and the fluid content of a fly was estimated to be 0.5 μl (see Materials and Methods).

The two lantibiotics tested, i.e., NAI-107 and nisin, showed good efficacy by effectively rescuing or delaying mortality of infected flies over a 96-h period (Fig. 2B and C). Treatment with NAI-107 at 3× MIC did not show a positive effect on the survival of 8325-4-infected flies. However, treatment with NAI-107 at 10× MIC rescued about 20 to 30% of flies (*P* < 0.001). NAI-107 treatment of control animals at 3× MIC and 10× MIC had no negative effect on survival (*P* = 0.62) (data not shown). We therefore tested NAI-107 at 100× MIC, and NAI-107 treatment at this concentration rescued more than 70% (*P* < 0.0001) of the infected flies, again without negative effects on survival of control animals (*P* = 0.62) (Fig. 2B). Compared to NAI-107, nisin showed differences in both efficacy and lethality in control animals. While nisin at 1× MIC delayed bacterial killing of flies (*P* < 0.001), it produced signs of lethal side effects in uninfected control animals injected with nisin at 1× MIC compared to VEH-injected control animals (*P* = 0.018) (Fig. 2C). A higher concentration of nisin (3× MIC) also rescued a considerable fraction of infected animals (*P* = 0.0002) but showed pronounced detrimental effects on the survival of control animals (*P* = 0.006). These adverse effects were exacerbated with nisin at 10× MIC, which resulted in the killing of 50% of control animals injected with nisin alone (*P* < 0.0001) and also resulted in increased mortality of infected flies (Fig. 2C). Therefore, nisin was not tested at 100× MIC.

In contrast to NAI-107 and nisin, the GN-4 peptide, which possesses good *in vitro* efficacy against S. aureus (Table 1) (41), did not rescue infected flies at 1× MIC and 3× MIC (*P* > 0.05) (data not shown). When applied at 10× MIC, GN-4 showed no detrimental effects on survival of flies (Fig. 2D). However, the results indicate that administration of this peptide to animals infected with bacteria may reduce survival, because a larger number of the animals treated with the peptide after infection died, although this was not statistically significant. The GN-4 peptoid showed pronounced negative effects on animal survival even at 1× MIC (Fig. 2E) and was therefore not subjected to further testing. The GN-2 peptide had effects similar to those of the GN-4 peptide, and the two GN-2 peptoids clearly showed adverse effects on survival in both control and infected animals (see Fig. S1 in the supplemental material). Injection of the HHC-9 and HHC-36 peptides in the absence of infection caused no obvious detrimental effects on survival. However, treatment with these peptides did not rescue infected flies but instead caused a moderate decrease in survival of infected animals that may indicate detrimental effects of peptides, although the results are somewhat ambiguous.

We also noted an adverse behavioral response that may indicate neurotoxicity in flies injected with high concentrations of nisin, GN-2, and GN-4, along with the peptoids, but not NAI-107. Animals reacted to injection with these compounds by being partially paralyzed for up to 10 h postinjection (data not shown). This paralysis was manifested not as complete immobilization but as uncoordinated movements and an inability to walk or fly.

### Treatment with nisin or NAI-107 reduces the immune response of S. aureus-infected Drosophila flies.

**FIG 3:**
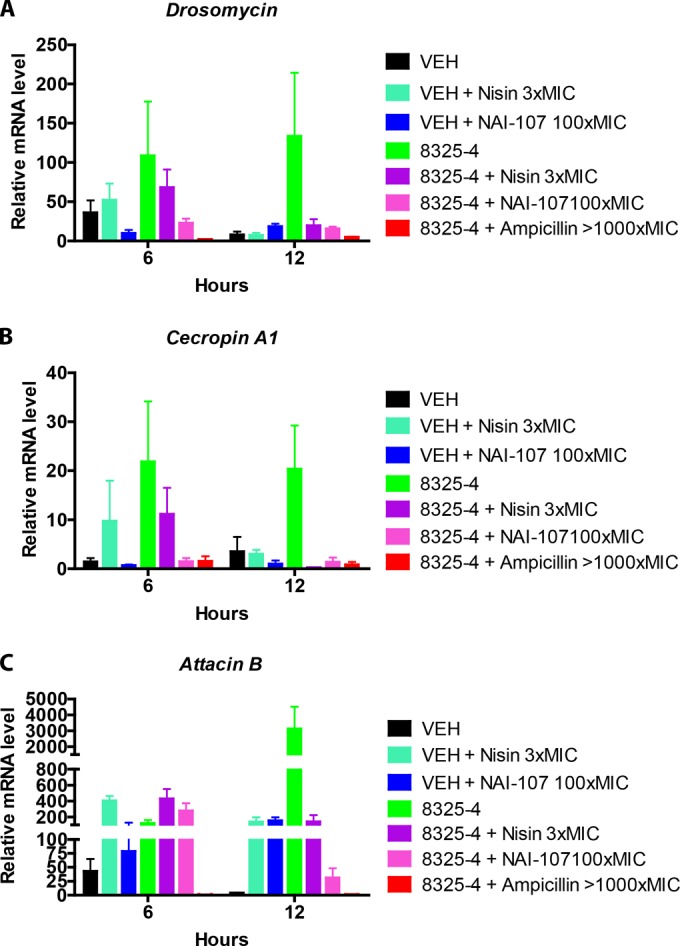
Induction of immune response genes. Drosomycin (A), *Cecropin A1* (B), and Attacin B (C) transcript levels were determined by qPCR. A noninfected control group was used as a reference for normal expression, and the average expression levels for this group were set at 1. Flies infected with S. aureus 8325-4 were sampled for qPCR analysis at 6 and 12 h postinfection. Drug treatment was performed at 3 h, and injection of vehicle (VEH) alone was used as a control. Due to the small sample size, statistical analysis was not performed. Error bars indicate standard errors (*n* = 3).

To further test the drug efficacy of the lantibiotics nisin and NAI-107 *in vivo*, we examined the immune responses of both treated and nontreated infected animals. We rationalized that infected animals treated with these compounds would mount less of an immune response provided that bacterial proliferation in the host was inhibited by the compounds. To test this, we used flies infected with S. aureus strain 8325-4. We administered NAI-107 at 100× MIC, while nisin, due to its detrimental side effect at high concentrations, was injected only at 3× MIC. Treatment of infected animals with ampicillin (>1,000× MIC) was included for comparison with an efficacious compound. Samples were taken in triplicate at 6 and 12 h postinfection, and uninfected flies served as controls. As a measure of the immune response, we analyzed the expression of the Drosomycin (*Drs*), *Cecropin A1* (*CecA1*), and Attacin-B (*AttB*) immunity genes, which have all been implicated in the immune response of Drosophila to infection by Gram-positive bacteria (49, 50). In general, we observed that animals that received any form of treatment had elevated transcription of immune response genes (Fig. 3), most likely because any injection into the animals damaged the tissue, thereby elevating the immune response. Moreover, it is highly plausible that injection of any protein-like structure will elicit some degree of immune response. Another general observation was the presence of higher expression levels of immune response genes in infected untreated animals than in animals treated with nisin and NAI-107.

The responses of the three immune pathway genes differed. Expression of *Drs* increased 30- to 180-fold within 6 h postinfection and remained at that level at 12 h postinfection (Fig. 3A). Treatment with NAI-107 and nisin decreased *Drs* expression approximately 10-fold relative to that in nontreated infected flies at 12 h postinfection. Expression of *CecA1* followed the same pattern as that observed for *Drs*, except that maximal induction was only around 20-fold (Fig. 3B). The *AttB* expression level was different, since gene expression was increased considerably in all flies injected with peptides and irrespective of a concurrent S. aureus infection (Fig. 3C). Because injection with VEH did not result in the same fold increase of *AttB* induction, we propose that the *AttB* gene was initially induced by either the pathogen or the administered peptides. The S. aureus infection further increased *AttB* expression, >1,000-fold relative to that of the control, at 12 h postinfection. Concurrent administration of nisin or NAI-107 reduced expression to the level observed for the peptides alone, or to an even lower level. Some compounds, including nisin, have previously been associated with immunomodulatory actions in mice (51). Consistent with this, our results indicate a moderate elevation in the expression of *Drs*, *CecA1*, and *AttB* in flies injected with nisin compared to VEH-injected control flies. However, whether this is due to true immunomodulatory action or because of the adverse side effects of nisin is unclear.

### NAI-107 efficiently rescues flies from infection with MRSA strain USA300.

**FIG 4:**
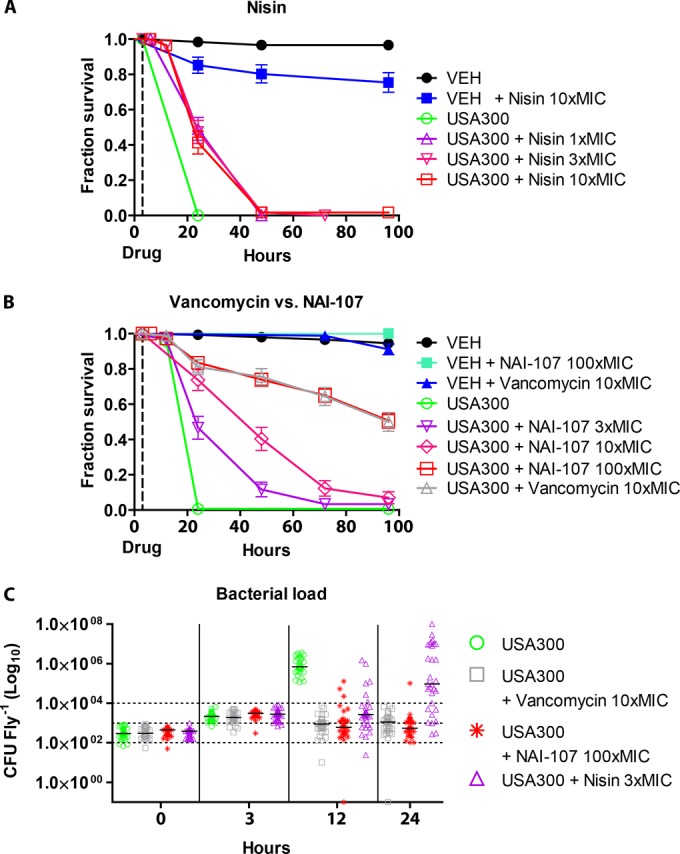
Efficacies of nisin and NAI-107 *in vivo* against S. aureus USA300. (A) Nisin prolonged the life span of infected flies at all concentrations. (B) NAI-107 rescued 50 to 60% of flies at 100× MIC (*P* < 0.001), similarly to vancomycin at 10× MIC. Antibiotics were injected at 3 h postinfection (dotted line). (C) Bacterial titers *in vivo* in flies of the different treatment groups. Treatment with antibiotics was given at 3 h postinfection, and bacterial titers at 3 h were determined prior to treatment.

We proceeded to evaluate the *in vivo* efficacy of lantibiotics relative to that of vancomycin in Drosophila flies infected with USA300. Flies were treated with nisin at 1× MIC, 3× MIC, and 10× MIC. Although nisin did not rescue flies over the duration of the experiment, it did delay mortality by doubling the mean survival time (*P* < 0.0001) at all concentrations tested (Fig. 4A). However, mortality was increased in the control group injected with nisin at 10× MIC relative to that of the VEH-injected control group (*P* = 0.0008). A single dose of NAI-107 at 100× MIC rescued 50 to 60% of USA300-infected animals over a 96-h period (*P* < 0.0001), equivalent to the survival found for vancomycin treatment of animals at 10× MIC (*P* = 0.94) (Fig. 4B). Positive effects on the survival of USA300-infected animals were also found at dosages of NAI-107 as low as 3× MIC (*P* < 0.0001). Similarly to NAI-107, vancomycin showed no adverse effect on survival of control animals at the concentrations tested here. The effect on survival of infected animals was further corroborated by the finding that *in vivo* treatment with vancomycin, nisin, or NAI-107 clearly disrupted the proliferation of bacteria inside the animals (Fig. 4C). By 24 h, the bacterial load for animals treated with NAI-107 dropped below the count seen after 3 h, the time at which the animals were treated. Our data also indicate that, by 24 h, NAI-107 was slightly more effective than vancomycin based on median values, although this was not statistically significant. However, treatment with NAI-107 or vancomycin significantly reduced the bacterial load in flies compared to that with nisin treatment after 24 h (*P* < 0.0001). Taken together, these results demonstrate that NAI-107 delays killing of Drosophila by systemic USA300 infections with an efficiency similar to that of vancomycin and with no changes to survival of control animals. This highlights the potential of NAI-107 as a candidate for systemically administered application.

## DISCUSSION

We used Drosophila as an infection model for testing the efficacy and adverse effects of peptides and peptoids. We examined several cationic antibacterial peptides and peptoids previously reported to have either *in vitro* or *in vivo* efficacy against S. aureus. Furthermore, two lantibiotics, nisin and NAI-107, were included. We found that both lantibiotics can delay or even rescue lethal injections with wild-type S. aureus 8325-4 and, more importantly, also those with the MRSA USA300 isolate.

We generally demonstrated that most of the amphipathic peptides and peptoids previously tested *in vitro* and/or *in vivo* against both Gram-negative and Gram-positive bacteria (41) had no positive effect on the survival of S. aureus-infected flies. These peptides are generally believed to work through electrostatic interactions with the negatively charged bacterial membrane (41, 42, 52, 53) and consequent pore formation, thereby disrupting the integrity of the bacterial membrane(s). This mechanism of action should exclude interaction with the more zwitterionic membranes of eukaryotic cells (52). The peptoids proved to be most detrimental to the flies, but the effect might simply be explained by the high concentrations of these molecules, as they had to be injected at high concentrations to reach the same multiple of the MIC as that for the corresponding peptide. Previous studies described that peptoids are hemolytic and cytotoxic *in vitro* at concentrations ranging from 100 to 170 μg ml^−1^ (45), yet our findings indicate that these compounds may have adverse effects *in vivo* even at lower concentrations (1× MIC [64 μg ml^−1^] for the GN-4 peptoid and 3× MIC [48 μg ml^−1^] for the GN-2 Ntrp_5–8_ peptoid). For the GN peptides, previous studies have shown that they are cytotoxic at levels of approximately 40 μg ml^−1^ (42, 45), which is largely consistent with most of our findings for comparing injected concentrations with the *in vitro* data. Although our data cannot exclude the possibility that the GN peptides and peptoids may be effective in mammalian models, the Drosophila *in vivo* data presented here do not support their use for whole-animal infections.

The cationic amphipathic HHC-9 and HHC-36 peptides had either no or a marked negative effect on the survival of infected flies. This contrasts with previous data showing that HHC-36 had low *in vivo* efficacy against S. aureus in a well-established mouse intraperitoneal (i.p.) model (44). However, Cherkasov et al. (44) tested the cytotoxicity of HHC-36 by hemolysis assay only, which makes it difficult to compare with our *in vivo* data. Furthermore, we observed that nisin reduced animal survival even at relatively low concentrations in our model. Previous *in vivo* findings from rat studies that utilized administration through oral dosing did not observe adverse effects of nisin (54). However, this might be explained by the fact that our study utilized injection into the circulatory system of whole animals, while rats were exposed through oral administration, which inevitably changes the bioavailability and potential adverse effects of a compound (55). Nisin has also been shown to be degraded by proteases in the digestive system (56). Perhaps nisin, because of its poor bioavailability and fast degradation (57), may be modified chemically to address these issues (19, 58), and in this context it would be important to know more about its potential adverse effects.

Although we expected the HHC compounds and nisin to be able to clear or delay infection in Drosophila, our results indicate that their injection into the circulation at high concentrations has negative effects on flies. The number of *in vivo* experiments performed previously for the analysis of systemic administration of HHC peptides and nisin is limited, and Drosophila has not been established as a model of infection directly comparable to other mammalian systems. Therefore, it is difficult to explain the observed differences. However, we believe that there is one important difference that should be considered between the *in vivo* studies performed with these peptides in mice and our work on Drosophila. In Drosophila, bacteria and peptides are delivered systemically into the circulation, while both are injected into the body cavity in the i.p. mouse model previously used to test the HHC peptides *in vivo* (44). It is not clear whether the peptides and bacteria enter the circulation in the i.p. mouse model, which may minimize adverse effects of these compounds and therefore not be predictive of negative effects in the whole animal. We argue that injection of peptides and bacteria into the open circulatory system of a fly provides access to more diverse tissues, which may be an advantage when it comes to identifying compounds with minimal toxicity and high efficacy during early phases of drug development. The open circulatory system of the fly may also make the fly model hypersensitive to adverse effects and explain the differences observed. We contemplate that hypersensitivity in the Drosophila model might actually be beneficial in early development for identification of candidates with good efficacy and a low risk of toxicity that will succeed during later stages of development. Late-stage failure in clinical trials has previously been reported as a problem in the development of peptide antibiotics (59). Although the potential for systemic application of nisin and most of the other molecules seems limited based on our findings, our data also reinforce the notion that nisin and these other molecules may have other therapeutic applications in clinical settings. These antibacterials may be developed further and optimized for topical usage, as was the case for the systemically toxic peptide antibiotic bacitracin, which has been highly successful in topical ointments (60, 61). However, it is important that adverse effects observed in Drosophila should not necessarily be considered a definite rejection of compounds, since these results may be used for further structure relationship studies and development of better compounds. Therefore, further studies are needed to address the intricate interactions of nisin with eukaryotic cell systems, especially since our data indicate possible adverse effects to the nervous system. Although the bacterial targets of nisin have been characterized (22, 62, 63), the interplay of nisin with other molecules of eukaryotic cells remains poorly understood.

To the best of our knowledge, Drosophila has not previously been used for testing of antibacterial peptide efficacy and toxicity. Drosophila does not allow for high-throughput screening of large drug libraries by injection, as this procedure is relatively labor-intensive compared to drug screening methodologies developed with the worm Caenorhabditis elegans (64). However, in contrast to C. elegans, our Drosophila model has the advantage that compounds that may be degraded during oral uptake can be injected into the circulation, which makes it suitable for testing of lead compounds. Therefore, Drosophila may prove important as an initial whole-animal model for identifying lead compounds with high efficacy and low toxicity, as classical toxicity screens usually involve hemolysis- and metabolic cell-based assays that do not recapitulate the complexity of a whole-animal system. To determine the usefulness of the Drosophila model of infection and whether it is hypersensitive, it will be important to determine its comparability to mammalian models and to develop strategies to measure actual drug concentrations in the fly hemolymph (blood). Based on our data presented here and the fact that Drosophila has proved to be a useful model for identifying other drugs, including anticancer therapeutics that are now used in the clinic (25, 65, 66), we believe that it has the potential to be an important model for antibacterial drug testing.

Additionally, our data enforce the notion that the lantibiotics remain of greatest interest for development of new therapeutics. As one of the best-studied lantibiotics (19), nisin recently gained new interest as a therapeutic because it was proven to be effective against MRSA (67, 68; this study), but it may require further toxicological studies. The newly discovered lantibiotic NAI-107 is currently undergoing preclinical studies, and it has already proven effective *in vivo* against MDR S. aureus (39, 69). NAI-107 delayed death due to infection at doses of around 10× MIC in Drosophila. Higher doses of NAI-107 resulted in remarkable *in vivo* efficacy, with no adverse effects. This is consistent with the previous finding that the effects of NAI-107 are concentration dependent (69). Nisin was clearly less potent than NAI-107 *in vivo*, although both compounds bind to lipid II (70) and rapidly kill bacteria.

In conclusion, we provide evidence for the use of Drosophila as a model for *in vivo* efficacy testing of antimicrobial peptides. Among the compounds we tested, the lantibiotic NAI-107 was superior to nisin but equivalent to vancomycin. Our data clearly show that infected flies can be rescued by treatment with certain antibacterial peptides. Importantly, the antibiotics (ampicillin and vancomycin) that are efficacious in the clinic are also efficacious in our model and do not produce any signs of adverse health effects. Furthermore, NAI-107, which shows efficacy in both i.p. and intravenous mammalian infection models (39, 69), shows high efficacy without adverse effects in our model. These results highlight that the Drosophila model is useful for evaluating the whole-animal efficacy of antibacterials. The Drosophila model presented here provides a cost-effective whole-animal system for development of lead antibacterial compounds with low toxicity and high efficacy.

## Supplementary Material

Supplemental material
