# Supplementary material for: The Lantibiotic NAI-107 Efficiently Rescues Drosophila melanogaster from Infection with Methicillin-Resistant Staphylococcus aureus USA300
Source: Antimicrob Agents Chemother. 2016 Aug 22;60(9):5427–36. doi: 10.1128/AAC.02965-15 (PMC4997821; doi:10.1128/AAC.02965-15)
Supplement: Supplemental material [file supp_60_9_5427__index.html]

The Lantibiotic NAI-107 Efficiently Rescues Drosophila melanogaster from Infection with Methicillin-Resistant Staphylococcus aureus USA300 — Supplemental material 

# The Lantibiotic NAI-107 Efficiently Rescues Drosophila melanogaster from Infection with Methicillin-Resistant Staphylococcus aureus USA300

## Supplemental material

- Supplemental file 1 -

  Fig. S1 and Table S1

  PDF, 427K
