# Supplementary material for: The Lantibiotic NAI-107 Efficiently Rescues Drosophila melanogaster from Infection with Methicillin-Resistant Staphylococcus aureus USA300
Source: Antimicrob Agents Chemother. 2016 Aug 22;60(9):5427–36. doi: 10.1128/AAC.02965-15 (PMC4997821; doi:10.1128/AAC.02965-15)
Supplement: Supplemental material [file AAC.02965-15_zac009165489so1.pdf]

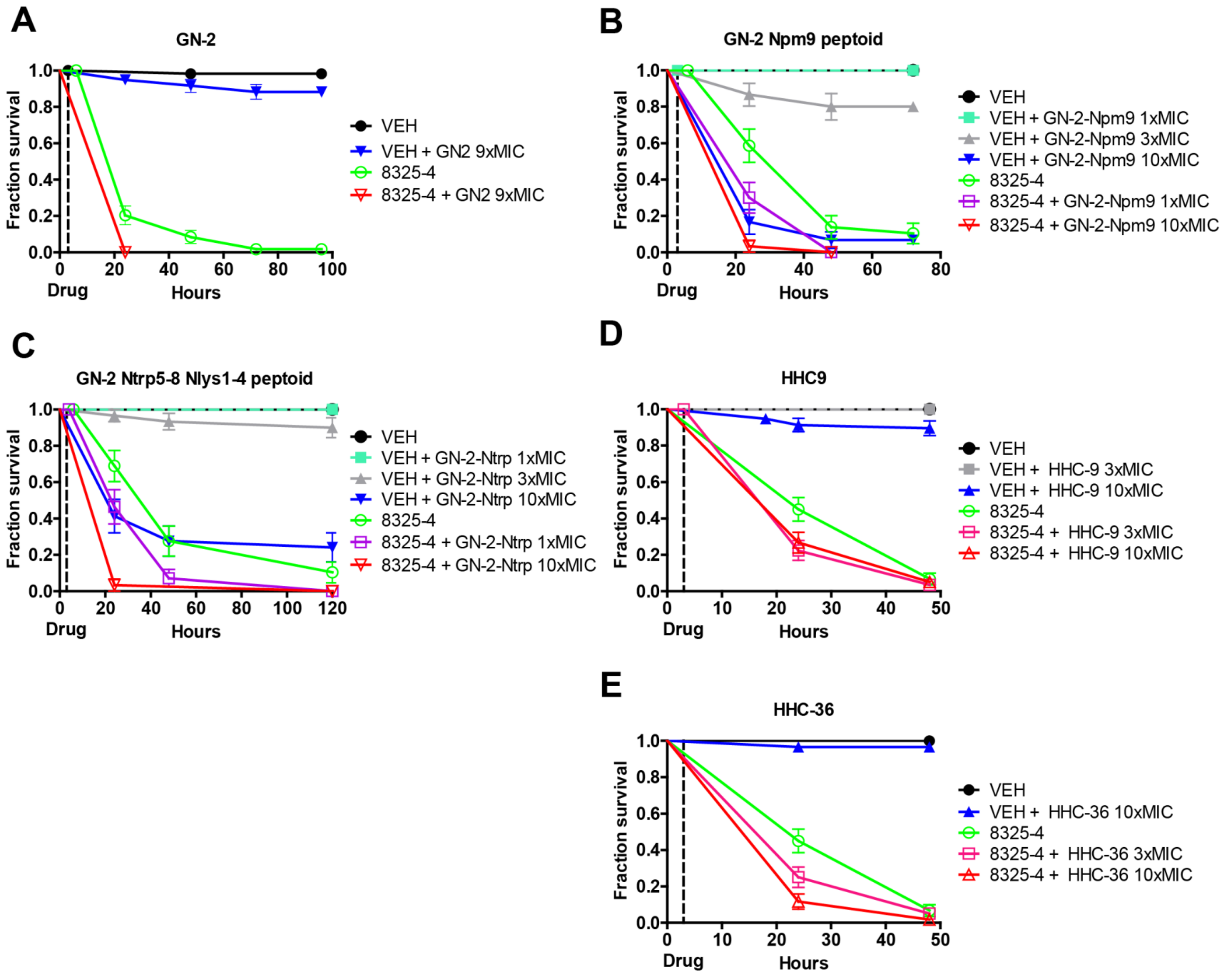

FIG S1 *In vivo* efficacy of compounds against *S. aureus* 8325-4 in a *Drosophila in vivo* model. (A-E) Survival graphs showing effect of different peptides and peptoids. Flies were counted at time points 0, 3, 6, 12, 24, 48 – 120 hours. Flies were either injected with vehicle (VEH) or isolate 8325-4 at time 0 hours and the + indicates treatment at time point 3 hours (dotted line). Flies were counted prior to injection with compound. Compound concentrations [C] are given as approximated concentration in animals.

TABLE S1 Oligonucleotide sequences.

Gene	Primer sequence
Cecropin A1: CecA1	F *CecA1*: 5`agctgggtggctgaagaa a-3`<br>R *CecA1*: 5`-attgtggcatcccgagtg-3`
Attacin-B: AttB	F *AttB*: 5`-cacaatgtggtgggtcagg-3`<br>R *AttB*: 5`-ggcaccatgaccagcatt-3`
Drosomycin: Drs	F *Drs*: 5`-gaggagggacgctccagt-3`<br>R *Drs*: 5`-ttagcatccttcgcaccag-3`
Ribosomal protein 23: Rpl23	F *Rpl23*: 5`-gacaacaccggagccaagaacc-3`<br>R *Rpl23*: 5`-gtttgcgctgccgaataaccac-3`
